# Stabilometric parameters analysis in children with visual disorder

**DOI:** 10.1186/1755-7682-7-1

**Published:** 2014-01-03

**Authors:** Paloma PC De Araújo, Oséas F De Moura Filho, Vitor E Valenti, Sophia Motta Gallo, Marcela R Camargo, Karina G Say, Renata S Marcomini, Gonçalves de Oliveira, Carlos BM Monteiro, Rubens Wajnsztejn, Luiz Carlos De Abreu

**Affiliations:** 1 Instituto de Ensino e Pesquisa em Fisioterapia Clínica de Saúde, Rua Vitório Orthiges Fernandes, 6123. CEP, Teresina 64073-505, PI, Brazil; 2 Departamento de Morfologia e Fisiologia, Faculdade de Medicina do ABC, Laboratório de Delineamento de Estudos e Escrita Científica, Av. Príncipe de Gales, Santo André 821. CEP: 09060-650, SP, Brazil; 3 Departamento de Fonoaudiologia, Faculdade de Filosofia e Ciências, Universidade Estadual Paulista, UNESP. Av. Higyno Muzzi Filho, Marília 737. CEP: 17525-900, SP, Brazil; 4 Departamento de Saúde Materno-infantil, Universidade de São Paulo, USP. Av. Dr. Arnaldo, São Paulo 715. CEP: 01246-904, SP, Brazil; 5 Laboratório de Análise do Movimento, Instituto de Ciências da Atividade Física e Esporte, Universidade Cruzeiro do Sul, Rua Galvão Bueno, São Paulo 868. CEP: 01506-000, SP, Brazil

**Keywords:** Postural balance, Visual disorders, Musculoskeletal system

## Abstract

**Background:**

Although postural changes were already reported in blind adults, no previous study has investigated postural stability in blind children. Moreover, there are few studies which used a stabilometric instrument to measure postural balance. In this study we evaluated stabilometric paramaters in blind children.

**Methods:**

We evaluated children between 7 to 12 years old, they were divided into two groups: Blind (n = 11) and age-matched control (n = 11) groups by using computerized stabilometry. The stabilometric examination was performed taking the gravity centers displacement of the individual projected into the platform. Thirthy seconds after the period in which this information was collected, the program defined a medium-pressure center, which was used to define x and y axes displacement and the distance between the pressure center and the platform center. Furthermore, the average sway rate and the body sway area were obtained by dividing the pressure center displacement and the time spent on the task; and by an ellipse function (95% percentille), respectively. Percentages of anterior, posterior, left and right feet weight also were calculated. Variables were compared by using the Student’s t test for unpaired data. Significance level was considered for p <0.05.

**Results:**

Displacement of the x axis (25.55 ± 9.851 vs. -3.545 ± 7.667; p <0.05) and average sway rate (19.18 ± 2.7 vs. -10.55 ± 1.003; p <0.001) were increased in the blind children group. Percentage of left foot weight was reduced (45.82 ± 2.017 vs. 52.36 ± 1.33; p <0.05) while percentage of right foot weight was increased (54.18 ± 2.17 vs. 47.64 ± 1.33; p <0.05) in blind children. Other variables did not show differences.

**Conclusions:**

Blind children present impaired stabilometric parameters.

## Background

Postural and balance control involves the management of the body position in order to keep it stable. Postural stability is the skill to maintain the body in balance; it is a measurement of postural oscillation [1,2]. Postural oscillation is defined as the constant deviation and correction of the gravity center position, as a result of a high gravity center and a small base support on orthostatic position, which puts the body in instable equilibrium [3,4].

Together with somatosensory and vestibular systems, the visual system processes information regarding the relative positions of body segments and the magnitude of the forces acting on the body which are necessary for the balance [5]. Without the adequate interaction among these systems, the organism may not acquire the ability to correctly explore and interact with the environment [6,7]. Among all sensory systems, humans tend to use primarily the vision [7].

Consider: Patients with proprioceptive impairment and visual disturbances may not maintain the posture effectively [8]. There are postural compensations acquired by blind individuals, such as the forward head and spinal abnormalities. Vision plays an important role in posture stabilization because it continuously provides information to the central nervous system with respect to the position and location of body segments in relation to the environment [9]. Visual acuity is defined as the eye ability to perceive the shape of objects. Patients with impaired vision present visual acuity lower than 6/60 in the best eye, whereas blindness refers to visual acuity lower than 3/60 in the best eye [10,11].

Children with impaired vision or other sensory disturbances have showed poor postural control and gait performance when they are compared with non-sensory impaired children [12-14]. Especially blind children were found to be unable to perform some aspects of gait (related to balance) as well as children without sensory disorders [13]. On the other hand, when normal children presented their vision occluded, they did not increase the stand sway [15], indicating that the possible key of balance disturbance in blind children is how their neuro-sensory-motor development occurs.

Although postural changes have been already evaluated in blind adults [16,17], in our knowledge, no previous studies have investigated postural stability in blind children using stabilometric parameters. In addition, stabilometry is an important and sensitive tool to measure postural stability and there are few studies which use this measurement instrument [10]. Therefore, in this study we proceeded to evaluate stabilometric parameters in blind children.

## Methods

### Study population

The study was conducted at the Piaui Association for Blind People (Associação de Cegos do Piauí – ACEP) and Nair Gonçalves School (Unidade Escolar Nair Gonçalves), where data were collected from blind children and control children, respectively. Both institutions are located in Teresina, Piauí. The study included 11 blind children – congenital and who lost their sight in childhood (with visual acuity lower than 3/60 in the best eye [12]) and 11 randomly selected age and gender-matched children without visual disorders who studied in Nair Gonçalves School. The parents of all children were informed about the research procedures during an interview where these procedures were fully explained and the study was approved by the Ethics Committee in Human Research of NOVAFAPI (Protocol Number 0242.0.043.000-9).

### Exclusion criteria

**Figure 1:**
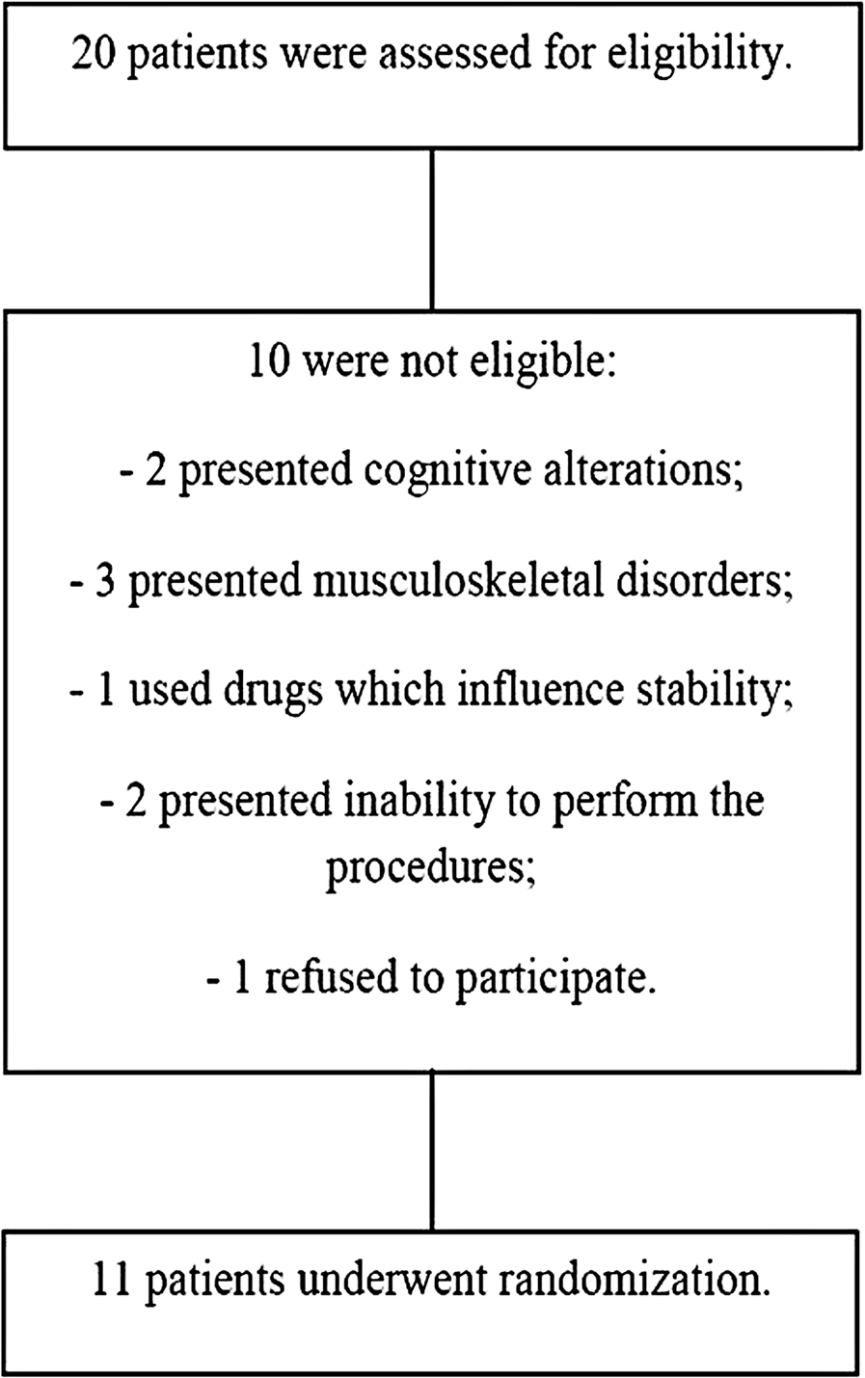
Multicentre trial flow diagram of blind children, including detailed information on the excluded participants.

We excluded children who presented cognitive alterations; musculoskeletal disorders which could affect their balance; and used drugs which could influence postural stability, especially alcohol, benzodiazepines and psychotropic substances, which modify certain parameters, inability to perform the experimental procedure; or those who spontaneously refused to participate. Therefore, eleven children remained in the study (Figure 1).

### Experimental procedures

Data collection was performed in the following order: filling the identification protocol (name, address, contact phone number, email address, sex, age), height measurement and stabilometric examination.

The stabilometric examination is performed using a rectangular shape unit divided into four quadrants, capable to perceive the weight force exerted on each quadrant. The stabilometric examination was performed taking the gravity centers displacement of the individual projected into the platform – pressure center (PC), created from averages of the anterior-posterior (x-axis-PC) and of medial-lateral (y-axis-PC) displacements. The distance between total PC and the force plate center was also calculated and it was called mean R. The average sway rate (ASR) and the body sway area (BSA) were obtained dividing the PC displacement and the time spent on the task; and by an ellipse function (95% percentile), respectively. Percentages of anterior, posterior, left and right feet weight were also calculated.

Data collection was initiated after subjects were stable at erect position on a force plate which was previously prepared. Subjects remained barefooted and positioned on the platform center with the feet facing forward, during 30s. The distance between the two feet considered the hip width and the longitudinal axis of the feet corresponded to the anterior-posterior axis of the force plate. The medial malleolus was aligned to the gap that separates the anterior and posterior sides of the platform. The arms remained relaxed and parallel to the body; the head was facing forward and the volunteer was asked to remain at this position.

### Statistical analysis

Data were presented as mean ± standard deviation of mean. The data normality was tested by Shapiro-Wilk’s normality test. As data were parametrically distributed, we used the Student’s t test for unpaired data in order to compare variables between blind and control groups (significance level was 5%, p <0.05). To reinforce that the sample size was enough, a statistical power of the test was performed adopting 5% as significance level (p <0.05). ASR, one of the most representative variable to postural control function [17,18] was selected to run the test. The statistical power analysis (using Cohen criteria [19]) has revealed that three subjects per group is sufficient to a statistical power of 96.57% 96.57% and a effect size ‘d’ of 4.23 – classified as very high index [20]. The statistical power analysis was processed using G*Power 3.0.10 software [21] and the data were processed using Prisma 5.0 software.

## Results

The average age of both groups was 9.818 ± 1.601 years old (p >0.05), ranging between 7 up to 12 year olds. In relation to children's height, the average was 137.546 ± 10.549 cm in the blind group, similar to the control group (138.546 ± 11.961 cm) (p >0.05). The mean weight was also similar between blind (32.013 ± 6.375 kg) and control (30.104 ± 7.874 kg) groups (p >0.05).

Table 1 presents the results about x-axis-PC, y-axis-PC, Mean R and ASR, which compares blind and randomly selected age and gender-matched children without visual disorders.

**Table 1:** Computed stabilometry variables in blind (n = 11) and control (n = 11) groups

**Groups**	**x-axis-PC (mm)**	**y-axis-PC (mm)**	**Mean R (mm)**	**ASR (mm/s)**
**Blind**	25.55 ± 9.851^a^	−30.91 ± 15.88	60.65 ± 9.426	19.18 ± 2.700^b^
**Control**	−3.545 ± 7.667	−32.45 ± 8.791	41.27 ± 6.272	10.55 ± 1.003

x-axis-PC: pressure center displacement in anterior-posterior orientation; y-axis-PC: pressure center displacement in mediolateral orientation; mean R: distance between total PC and the force plate center; ASR: average sway rate – obtained by dividing the PC displacement and the time spent on the task. Non-paired Student t test – ^a^p < 0.05: compared to control group; ^b^p < 0.001: compared to control group.

Table 2 displays the computerized stabilometry variables regarding percentage of left and right feet weight and percentage of anterior and posterior weight.

**Table 2:** Mean and standard deviation of computed stabilometry variables in blind (n = 11) and control (n = 11) groups

**Groups**	**Left feet**	**Right feet**	**Anterior**	**Posterior**
	**(% of weight)**	**(% of weight)**	**(% of weight)**	**(% of weight)**
**Blind**	45.82 ± 2.017^a^	54.18 ± 2.017^a^	41.82 ± 3.835	58.18 ± 3.835
**Control**	52.36 ± 1.330	47.64 ± 1.330	40.73 ± 2.115	59.27 ± 2.115

Non-paired Student t test: ^a^p < 0.05: compared to control group.

**Figure 2:**
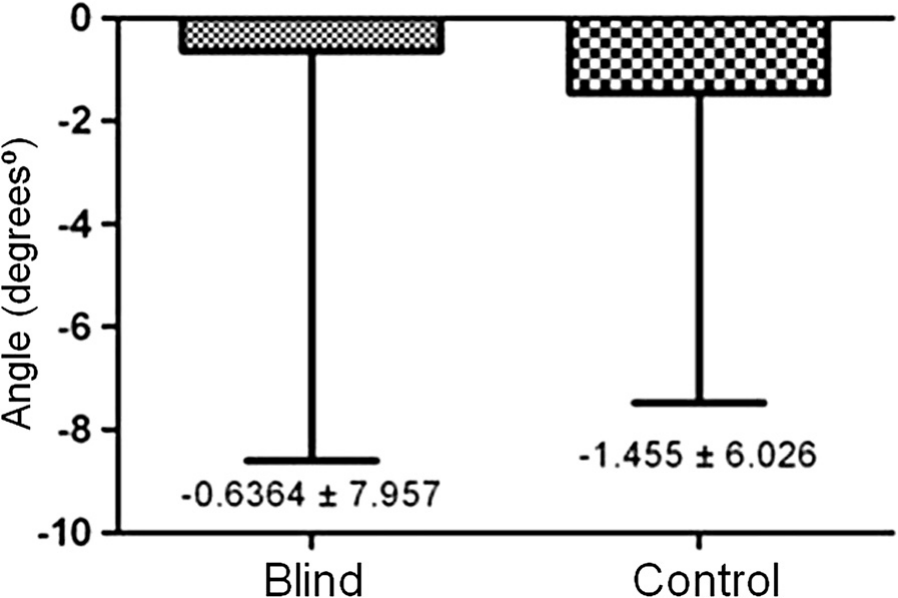
Dispersion angle variability in blind and control groups.

**Figure 3:**
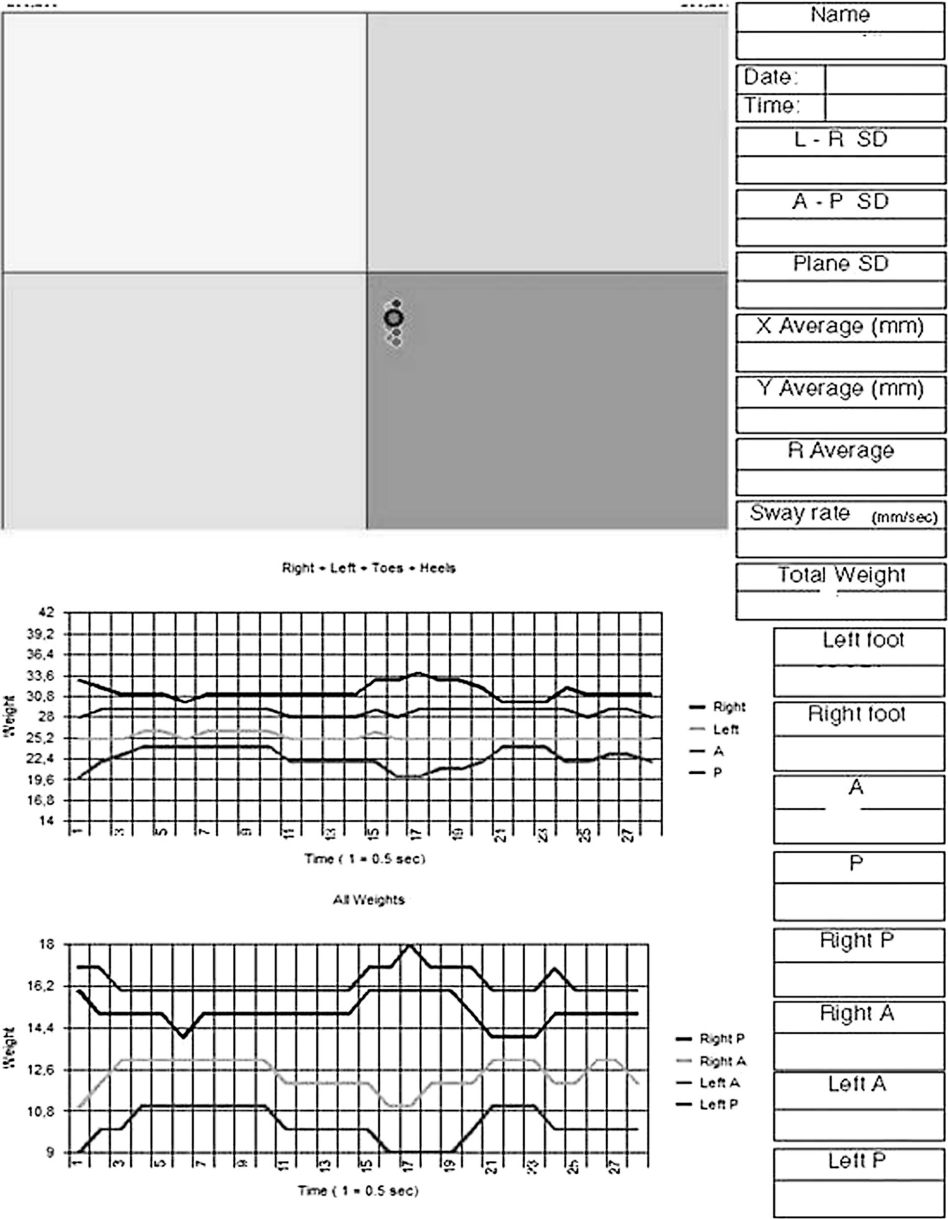
**Stabilometry examination result model according to *Posture Analyzer *software.** Midot Medical Technology, 2009.

In relation to postural oscillation, BSA has demonstrated high angle dispersion; however, no significant differences between blind and control groups were observed (Figure 2). Figure 3 presents the stabilometry examination result model according to *Posture Analyzer* software.

## Discussion

This investigation was undertaken to evaluate stabilometric parameters in blind children. As main results we found that the group composed by blind children presented: higher x-axis-PC displacement; higher ASR; lower percentage of left feet weight; and higher percentage of right feet weight. Our results indicate that blind children present impaired postural stability.

Previous experiments indicate that children between 7 to 10 year olds the child begin to present similarities of gait and balance to adults [22]. That is the reason for our group selection, which ranged between seven and 12 years old (9.818 ± 1.601 years). It facilitates the comprehension of our stabilometric results, since there are no normality standards for the infantile population.

The higher x-axis-PC displacement (25.55 ± 9.851 vs. -3.545 ± 7.667 mm; p <0.05), performed by blind children, shows an increased medial-lateral (right-left) oscillation. Such variation was not reported in the y-axis and, therefore, it did not reflect in anterior-posterior oscillation changes. The medial-lateral oscillation is related with hip strategy of postural maintenance, in which the hip muscles are recruited to avoid the individual fall down [23]. In this way, our findings agree with Ray et al. [24] who reported that adults with vision loss use more hip strategy to maintain their balance than non impairment people.

Our study showed no differences between blind and control groups regarding mean R, although the blind group tended to present higher values (provided mainly by x-axis-PC displacement), it did not reach statistical significance. Due to this variable is consider to be an absolute position measure, even that descriptive, it is not broadly representative as other postural control variables, like ASR for example [25].

The ASR was increased in the blind group compared to the control group (19.18 ± 2.7 versus 10.55 ± 1.003 mm/s; p <0.001). These data suggest the difficulty of blind children to stabilize their posture, given the discrepancy of the x-axis-PC displacements. Jeka et al. [17] performed a study with healthy adults which demonstrated that the change of postural behavior provided by experimental conditions, i.e. no vision (eyes closed), can be attributed, mainly, to the accurate velocity information loss. Therefore, if this information is altered, it alone can reflect negative impacts on postural control.

In relation to BSA, we reported similar behavior between both groups and large discrepancies between individuals of the same group, which probably occurred because it is an age at which motor aptitudes still are not developed. The blind group tended to impose weight to the right side, while the control group tended to impose weight to the left side. It was not evaluated the question of dominance in our research. Maybe if it was investigated, it may explain the weight distribution behavior in left and right legs. The trend towards higher weight on the posterior side observed in both groups, with consequent lower discharge weight on the anterior side, may be attributed to the stage of motor development and postural characteristic of the children’s age [22,26,27].

The comparative stabilometric analysis between blind and control groups revealed high instability degree of left-right PC, high oscillation average rate and tendency to discharge the postural weight into the posterior part of the body in blind children compared to children without visual disorders at the same age.

People, in their daily tasks, need to recruit information from all sensory inputs (somatosensory, vestibular and visual) to execute them successfully with less energy expenditure as possible [28,29]. Depending on the task demands, people reweight these sensory inputs, preferring one or other input more emphatically [30-32]. This reweighting is maturated during the childhood and after the age of 10 or 11 years old the performance is similar to adults [22]. However, blind children could not rely on visual input, therefore their sensory reweight is altered and our results show this alteration provides negative consequences on the oscillation velocity of the body. Thus, it is important to investigate how the reweight of sensory inputs is developed in blind children, looking for similarities and differences with non impairment children.

Although all blind children were registered at the ACEP evaluated, which represents nearly the total of blind children living in Teresina, since this is the reference center of attention for such disability, the sample used in our investigation can be considered small. Nevertheless, the statistical power of the test provided us support to use it, even with this amount of subjects.

In conclusion, blind children present impaired stabilometric parameters compared to children without vision disorders.

## Competing interests

The authors declare that they have no competing interests.

## Authors’ contributions

PPCA, OFMF, VEV, SMG, MRC, KGS, RSM, AGO, CBMM, RW and LCA participated in the revision of the manuscript. PPCA, OFMF, VEV and LCA determined the design, interpreted the text and drafted the manuscript, CBMM, AGO, RW, RSM, MRC, SMG and KGS drafted the manuscript. All authors read and gave final approval for the version submitted for publication.
